# Building International Genomics Collaboration for Global Health Security

**DOI:** 10.3389/fpubh.2015.00264

**Published:** 2015-12-07

**Authors:** Helen H. Cui, Tracy Erkkila, Patrick S. G. Chain, Momchilo Vuyisich

**Affiliations:** ^1^Bioscience Division, Los Alamos National Laboratory, Los Alamos, NM, USA

**Keywords:** genomics, next-generation sequencing, international collaboration, pathogen detection, global health, one health, bioinformatics, capability development

## Abstract

Genome science and technologies are transforming life sciences globally in many ways and becoming a highly desirable area for international collaboration to strengthen global health. The Genome Science Program at the Los Alamos National Laboratory is leveraging a long history of expertise in genomics research to assist multiple partner nations in advancing their genomics and bioinformatics capabilities. The capability development objectives focus on providing a molecular genomics-based scientific approach for pathogen detection, characterization, and biosurveillance applications. The general approaches include introduction of basic principles in genomics technologies, training on laboratory methodologies and bioinformatic analysis of resulting data, procurement, and installation of next-generation sequencing instruments, establishing bioinformatics software capabilities, and exploring collaborative applications of the genomics capabilities in public health. Genome centers have been established with public health and research institutions in the Republic of Georgia, Kingdom of Jordan, Uganda, and Gabon; broader collaborations in genomics applications have also been developed with research institutions in many other countries.

## Background and Introduction

In early 2010, the prominent general scientific society in the US, the American Association for the Advancement of Science (AAAS), recognized that international scientific collaboration is critical to addressing complex societal challenges in health, agriculture, environment, energy, and global security, and organized a series of four conferences under the common theme of “International Engagement: Responsible Bioscience for a Safe and Secure Society.” In a period of 2 years, these conferences examined the critical issues and explored the potentially sustainable approaches for collaboration between the scientists in the US and other countries, with the focus on the broader Middle Eastern and North African region. The major themes resulting from these conferences revealed a clear desire for cooperative research and development with responsible scientific practice and progressive action to enhance the infectious disease surveillance under the One Health concept (1). Although the AAAS conference series was focused on one region, the concept derived from the meeting and approaches that can be taken are undoubtedly applicable to many other countries and regions. Successful and sustainable partnerships that build upon mutual interests, complementary capabilities, and supporting infrastructure are essential to addressing complex global challenges, such as approaching the One Health concept. Scientists with interest and resources to initiate and maintain partnerships with colleagues in other countries, and who are able to overcome existing barriers to collaboration, serve as the foundation for successful international cooperation.

Reducing global health security risk from the spread of dangerous infectious diseases, whether natural or manmade, is a shared priority among the worldwide public health communities. It has also become an overarching objective for international cooperative biothreat reduction and scientific engagement efforts. Engaging with and empowering infectious disease detection and surveillance capabilities in the partner countries enable a global network to reduce risk and enhance compliance with international guidelines, such as the International Health Regulations (from the World Health Organization in 2005) and those by World Organization for Animal Health (OIE). Extensive international collaborations have been developed to address global health security challenges, with activities, such as bioethics discussion, responsive scientific conduct, biorisk management, and field epidemiology, which have resulted in a positive global impact.

A well-suited scientific approach complementary to the above activities is next-generation sequencing-enabled genomics research for global health security applications. Genomics is a relatively new scientific discipline but is fundamental to many approaches to health security. Aided by highly automated Next-Generation DNA Sequencing (NGS) technologies, the field has been advancing dramatically, both in the depth of understanding genome structure and function of living organisms, and in the breadth of applications in areas, such as medicine (disease mechanisms, diagnostics, and therapeutics) and agriculture (2, 3). Using high-throughput DNA sequencing for pathogen detection during an infectious disease outbreak or for biosurveillance was previously slow, cost prohibitive, and required in-depth training and expertise. In the past decade, due to the advancement of highly automated instrument platforms, streamlined operational procedures and protocols, available reagent kits, and data analysis pipelines (4), NGS has been in effect democratized, expanding from central sequencing laboratories to individual institutions. As a result, genomics has become a highly desirable area that inspires broad international collaborations targeting diverse applications (5). Examples of global health security related applications include infectious disease diagnosis, previously unknown pathogens identification, sample archive characterization, and biosurveillance. Even though NGS technology has made high-throughput DNA sequencing more accessible, the existence of the automated instruments and streamlined procedures cannot replace the in-depth knowledge and expertise to use the instruments with high proficiency and accuracy; nor can the analytic algorithms analyze the experimental outcomes with precise interpretation. Without specialized training and technical expertise, these technology advancements cannot be fully utilized.

In support of the overarching scientific engagement objectives, the Genome Science Program at the Los Alamos National Laboratory (LANL) has been leveraging our own capabilities to provide support to a growing number of partner countries on four continents in developing molecular genomic-based pathogen detection and characterization capabilities. We approach such development by first understanding partner country needs and gaps in building genomics and bioinformatics capacities. This is followed by scientific and technical training, facility building, and dissemination of pipelines and processes for microorganism genotypic characterization. Continuous subject matter expertise reachback support is provided to the collaborators. Our efforts in these areas enable the education of the next generation of life scientists in the partner countries, providing a robust foundation in genomic science through didactic and practical instruction, and the required technical infrastructure for genomics research by integrating sequencing and analytic capabilities.

While these genomics capabilities are being developed, the collaborators start engaging in scientific collaborations utilizing NGS and other molecular techniques, such as real-time PCR and immunoassays. By applying these methods and correlating the findings, we aim to better understand emerging infectious diseases within the partner countries that are of global concern. The collaboration efforts will not only benefit the host countries and regions with state-of-the-art life science methods and technologies but also build a trusted international network with a shared passion in addressing global emerging infectious disease challenges. Such networks provide the potential for sharing resources, which is essential for approaching the One Health objective and reducing health threats globally.

## Strategy and Approach

Our international genomics development and collaboration efforts have been built upon prior engagement activities carried out by other institutions and sponsored by various donors. We initially sought collaborations with countries that have been partners with our sponsors for several years, such as the Republic of Georgia and the Kingdom of Jordan. Many of the collaborating institutions already have well-established capabilities in molecular diagnostics and genetic analysis, and hold principle responsibilities in public and veterinary health in their respective countries. To effectively achieve the scientific engagement objectives, we have developed a phased strategy for establishing genomics laboratories in partner countries, followed by continuous technical support and cooperative scientific research to address some of the pressing public and veterinary health challenges.

### Phased Approaches for Capability Building

Our phased approach for establishing genomics centers with partner countries provides both the starting momentum to build a capability and flexibility that is responsive to the evolution of technologies and business practices, which reduces risks in achieving sustainability. The four phases can be summarized as: (1) scientific engagement evaluation, (2) technical strategy and development planning, (3) infrastructure and technical capability building, and (4) instrument operation and bioinformatics training. One most critical consideration throughout all phases is the sustainability development. Depending on the existing capabilities and infrastructure readiness, we do not necessarily go through all phases with any given partner institution.

**Figure 1:**
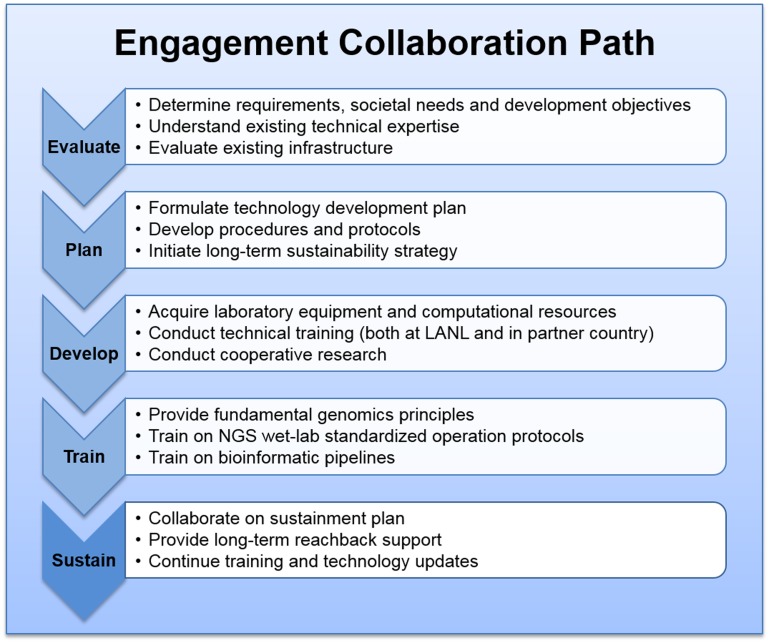
**Phased approach, for NGS-based scientific engagement development**.

The phased approach to engagement and sustainment is shown in Figure 1.

#### Phase I, Scientific Engagement Evaluation and Requirement Analysis

In order to provide meaningful assistance to our partner countries and their particular institutions, it is essential to understand host country public health challenges and requirements, and to carefully consider what a genomics capability can accomplish to address their specific challenges. We first work directly with host nation scientists and leadership to understand existing capabilities and expertise in pathogen detection and characterization, urgent public and veterinary health issues, and to assess the technologies and capabilities compatible with genomics and bioinformatics. Essential areas of consideration include: host country objectives in genomic science and technology, desired near- and long-term outcomes, type and availability of samples to be processed, anticipated throughput and turnaround, potential future resources, anticipated implementation timeline, and a long-term sustainability vision. Other assessment areas include the existing technical expertise in laboratory operation and bioinformatics, laboratory infrastructure, computational infrastructure, and administrative skills. An initial development strategy is formulated based on a requirement analysis for the engagement partner to achieve full operational capability, including the approach to narrowing the gaps between the desirable end goals and the current status of these resources.

Direct interactions with prominent regional life scientists, medical, public health, and agriculture professionals provide the collaborators with unique opportunities to identify areas for advancement that would significantly impact the science in the region or in the country; this allows us to build trust for a long-term collaborative relationship. The availability of advanced genomic science capabilities meets the foundational need that would impact almost all areas of the life sciences and presents opportunities for technology and economic development. During this phase, we start deliberating a long-term sustainability plan, based on the initial observations and development objectives.

#### Phase II, Technical Strategy and Development Planning

Once the requirements and objectives are defined, we begin formulating the technology strategy and development plan for a given institution to reach the target objectives. Planning includes personnel professional development, infrastructure preparation, NGS instrument acquisition, and initiating collaboration logistics including sample transportation and information sharing procedures. A technical action plan is generated that provides a framework of recommended protocols, workflows, and required training. In addition, the action plan provides recommended paths to address the gaps and challenges in the existing laboratory equipment, facility, and computational resources. We also generate a training plan to outline the tasks required for laboratory staff to obtain proficiency using specific operational protocols, pipelines, and processes. This includes sample preparation and management, instrument operation, sequence data analysis, and initial interpretation for biological significance.

During this phase, we also introduce fundamental principles and basic techniques of molecular- and genomic-based approaches for pathogen and infectious disease detection and characterization, and assist partner countries to further define priorities and needs for enhancing these capabilities. Initial training in these topics is provided either at LANL and/or at the partner site.

#### Phase III, Infrastructure and Technical Capability Development

The engagement activities subsequently progress into preparation of the NGS laboratory facility, acquisition of laboratory instruments and computational equipment, dissemination of standardized operational protocols, and technical training for proficiency building. The engagement collaborators work closely to design and configure appropriate laboratory space and bioinformatics systems at the partner facility. In this phase, careful consideration of local constraints, logistics, and experience available at the engagement partner site are critical.

The steps in this phase include

1. *Wet-lab and informatics hardware specification and laboratory setup*. The specification is based on the existing facility infrastructure, equipment, expertise and target goals. It includes wet-lab operational protocols and bioinformatics systems required to accomplish the development objectives. Laboratory equipment required for various wet-lab processes, and informatics hardware and software to process data and perform analysis are specified and acquisition is initiated. In some cases, the acquisition is handled by LANL, and in other cases by a third-party integrating contractor. Laboratory setup is performed by a combination of vendors, partner staff, and LANL staff. Good laboratory practice (GLP) is used as a guiding principle to ensure high quality experimental output, minimize potential cross-contamination, and protect the laboratory personnel.
2. *Deploy wet-lab processes*. We work with engagement partners to deploy standardized wet-lab protocols and processes. Example protocols include DNA library preparation for bacterial isolate sequencing, RNA library preparation for viral sample sequencing, targeted microbial sequencing protocols, whole genome sequencing (WGS), and general Illumina MiSeq platform sequencing procedures. Implementation of GLP is stressed during this phase, and training in GLP is delivered throughout all relevant phases.
3. *Deploy informatics pipelines*. Informatics pipelines coordinated with wet-lab protocols are provided to the partner facility. Examples include initial sequencing data quality control and reporting, data quality treatment, genomic assembly, reference-based assembly, and metagenomic analysis.

#### Phase IV, Training on Instrument Operation and Bioinformatics Processing

A strong foundation built through training is a vital component of the entire engagement activity. The collaborators work closely to coordinate training sessions in both the US and on-site in the host country facility. The primary goal is to instill a strong foundation in both scientific principles and operational processes during the exchange. On-site training focuses on development and implementation of sample preparation, sequencing library generation, genome sequencing, and sequencing data analysis procedures in compliance with GLP regulations. Great attention is paid to hands-on troubleshooting, and additional training is provided as needed. Training in this phase is an avenue to disseminate best biosafety and biosecurity practices.

Throughout the exchange and following period, the US scientists monitor partner performance and provide feedback to the sponsors and partner country scientists. Bioinformatics reachback assistance is provided to support current mission and long-term development of sustainable approaches. Assistance is also provided to the partners in the development and execution of research projects that utilizes the capability. Updating or expanding protocols and processes and providing new versions to the engagement partners are a continuous practice.

### Sustainability Development

The sustained utilization of the new genomics capability and continued service to society is the ultimate goal for the genomics centers being developed. To support sustainable development, we assist the institutions in identifying research and operational areas, such as training their fellow scientists and public health practitioners, conducting hypothesis-driven research, and providing services to community. The basic research, applied research, and operational areas that will benefit from advanced genomics science are extremely broad, virtually in all life sciences and related fields of practice. Priorities are identified based on research activities, public health responsibilities, and perceived societal significance. These priority areas include emerging or unknown pathogenic microorganism identification, molecular epidemiology, phylogenetic characterization of infectious agents, and infectious disease surveillance. In addition, genomics-assisted design and development of new diagnostic signatures and assays, and their applications in veterinary and agricultural practices have become important areas of engagement.

After deploying genomics technologies at a partner site and delivering initial training, successful capability establishment at the host site typically requires on going assistance. LANL provides reachback support to the engagement partners, by delivering remote, near real-time technical advice on wet-lab, informatics, and data analysis. Laboratory support includes advice on sample preparation procedures and protocols, and recommendation of specific reagents or kits to use. Informatics-focused reachback support can be especially important for some partners, as they may have limited existing informatics capability to draw on. Reachback on data analysis is very common, and can usually be a point of collaboration, from sequencing data quality evaluation to interpreting biological significance. Internet connectivity is very important for these purposes, and can sometimes be the rate-limiting factor.

Scientists at partner institutions are regularly invited to attend and contribute to the annual Sequencing, Finishing, and Analysis in the Future (SFAF) meetings that LANL has hosted for the past decade [SFAF (6)]. This conference provides an opportunity for the engagement partners to gain up-to-date knowledge on new techniques and technologies, and to present their own research results. Since 2013, LANL has hosted annual NGS training workshop in Los Alamos for engagement partners. The workshop includes principles of NGS and hands-on training in both laboratory techniques and bioinformatics. These opportunities provide avenues to broaden expertise and our collaborations with international partners.

## Results

We have successfully established genome centers with partner institutions in four different countries: Republic of Georgia, Kingdom of Jordan, Uganda, and Gabon, and developed extensive genomic research collaboration with several other countries. These genome centers are provided with a standardized sequencing platform, standard operating protocols and GLP training, and bioinformatics analysis tools and associated computational hardware.

### Standardized Platforms and Processes

**Figure 2:**
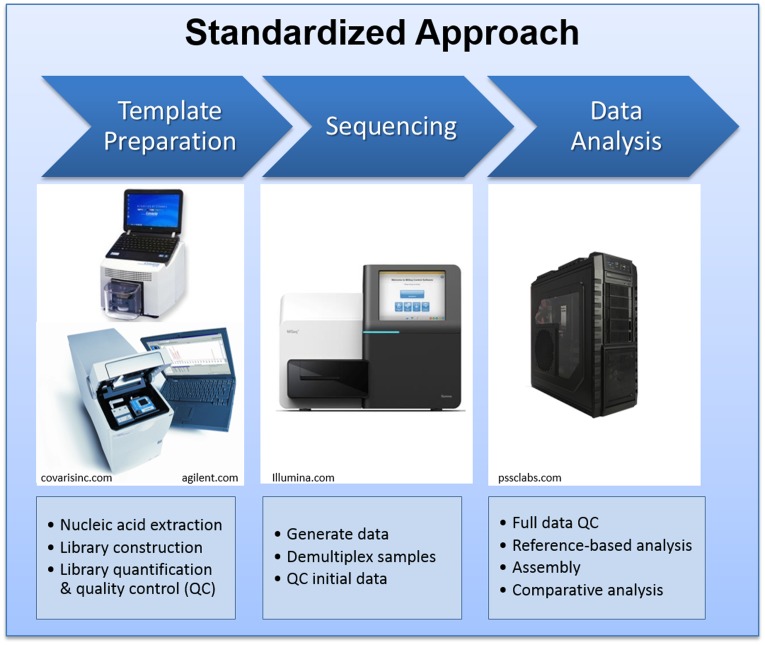
**The standard NGS laboratory and bioinformatics pipeline setting deployed to the partner laboratories**.

At LANL, we have extensive experience with most NGS platforms. During the past decade, we have acquired every major NGS instrument to conduct scientific research and to provide sequencing service and training to our collaborators. The Illumina MiSeq instrument was selected as the sequencing platform of choice for the international genomics engagement activities for its compact physical size, accompanying reagent kits, standardized operation procedures, high quality data output, and broad applications (4, 7). We have developed and disseminated standardized protocols for sample preparation, quality control, sequencing, and data management protocols. The standardized protocols require a few other supporting laboratory instruments for DNA fragmentation, quality control, quantification of DNA fragments, and quantitative PCR, which are also provided to our partner facilities. The general process flow is summarized in Figure 2.

Standard computer software and hardware have also been specified to simplify installation and ongoing support. For computational resources, the CLC Genomics Workbench (Qiagen) was selected from several commercial options as an analysis and visualization tool. This software package provides an integrated environment with most types of tools necessary for our partners to analyze sequencing data generated by MiSeq. In-house developed bioinformatics tools are also disseminated to the partner laboratories. The LANL developed EDGE Bioinformatics (8) is an analytic tool that integrates a selection of open source tools accessed by a web-based interface. EDGE has been deployed to several of our partner facilities and has proven to be an easy-to-use tool for rapid analysis of NGS data, requiring little specialized bioinformatics expertise. The computational hardware provided to our partners is fully sufficient to process sequencing data generated by the MiSeq platform at the partner’s site. This approach to deploying and training on a repertoire of technology and equipment has enabled us to franchise genomics centers with standardized procedures for our partners. The training and support we can provide is greatly enabled by this standardization.

### Current Collaborations

**Figure 3:**
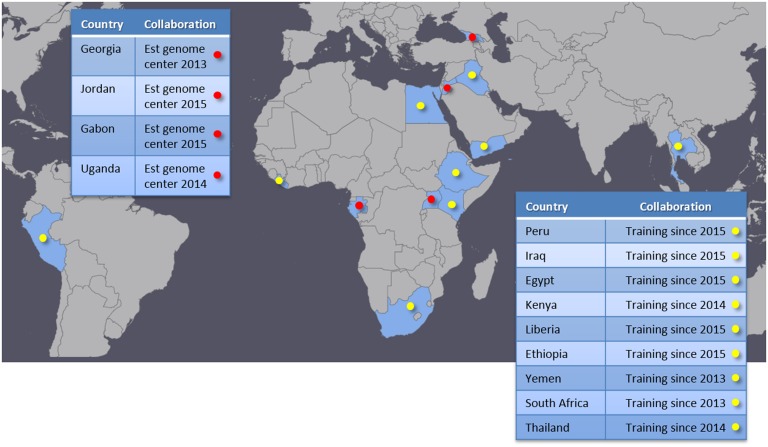
**Partner countries**. Red marks indicate genome centers established by our program. Yellow marks countries that are in collaborations focusing on training and research.

We have successfully established genome centers with four partner institutions in Georgia, Jordan, Uganda, and Gabon (Figure 3). These genome centers are provided with a standardized sequencing platform, standard operation and GLP protocols, and bioinformatics analysis tools and associated computational hardware. We travel to each site and assist with setup and configuration for laboratory equipment and computational resources. On-site training for both laboratory and bioinformatics are also provided during these visits. We typically guide the partners to perform one or more sequencing runs on the newly provided equipment while we are on-site. A brief description of each genome center is provided in Section “Established Genome Centers” below. In addition to establishing these genome centers, we provide training and engage in collaborative research with other institutions that either already have NGS capability or are on a path to acquire it. This type of collaboration has taken place in nine different countries (Figure 3), and allows the partners to broaden international collaboration networks and to exercise and enhance local capabilities. These collaborations bring together complementary skill sets and resources, benefiting all participating parties and paving a good path to improved global health.

### Established Genome Centers

#### Republic of Georgia: Genomics Center at the National Center for Disease Control and Public Health

The National Center for Disease Control and Public Health of Georgia (NCDC) was originally established in 1937 as the Anti-Plague Station, investigating species and the spread of *Yersinia pestis* in Georgia. After a tularemia outbreak in Southern Georgia in 1946, the institutional mission scope was expanded to include the investigation of tularemia and subsequently other infectious diseases, such as anthrax. The institution has since become the leading epidemiological control organization in Georgia, and later established the national reference laboratory now known as the R. G. Lugar Center for Public Health Research in 2013.

The genome center at Georgian NCDC was established in 2012. Extensive training took place for the Georgian scientists at both LANL and NCDC on NGS sample preparation protocols, MiSeq operations, and bioinformatics analysis of sequencing data. Reachback support in the form of teleconference calls, emails, and remote logins has been delivered to the Georgians on a regular basis. Today, the NCDC genome center is fully functional and more than 10 staffs have been trained and are regularly using the capabilities to conduct scientific research in pathogen virulence characterization and biosurveillance for infectious diseases. LANL and NCDC scientists have also embarked on joint research projects utilizing the new NGS capability. An example of research activities is highlighted in Section “Initial Cooperative Research Highlights,” and a new project has been initiated as a result of a competitive proposal process.

#### Kingdom of Jordan: Jordan University of Science and Technology

The Princess Haya Biotechnology Center (PHBC) at the Jordan University of Science and Technology (JUST) was established in 2005. The Center represents the state-of-the-art biotechnology in clinical applications, supported by Princess Haya Bint Al Hussein, with a primary responsibility directed toward supporting the Jordanian medical and scientific communities. It is hosted at the King Abdullah University Hospital and is equipped with modern facilities for carrying out research, diagnostic laboratory testing and training in areas of genomics, proteomics, metabolomics, hematology, and other clinical areas.

In 2012, LANL initiated collaborations with the PHBC to develop an NGS genomics capability. Applying the phased approach detailed in Section “STRATEGY AND APPROACH,” a functional NGS facility for genomics research and training was established in early 2015. Leveraging our experience with the Georgian project, we specified laboratory equipment and computational resources to match the capability target, level of PHBC staff expertise, and existing PHBC laboratory resources. During 2013 through 2015, extensive training and traveling took place for JUST staff to receive GLP, laboratory protocols, and bioinformatics analysis trainings, at both LANL and JUST. Currently, joint research projects are being planned to exercise the genomics capability and further train JUST staff.

#### Uganda: The Uganda Virus Research Institute

Uganda Virus Research Institute (UVRI) is a Ugandan national reference laboratory for various viral diseases including hemorrhagic fevers, HIV, and Influenza. An NGS capability based on the MiSeq platform is being incorporated into laboratory operations at UVRI, especially during epidemic outbreak investigations for enhanced pathogen detection.

In August 2013, LANL staff traveled to Uganda for the initial evaluation of UVRI facilities and expertise, the Phase I in our overall approach. New construction at UVRI was beginning on the space that would become the home of the genome center. Recommendations on infrastructure details as a fully functional genomics laboratory were quickly provided and implemented. Detailed planning for the genome center development completed in 2013. During 2014, facility construction was completed and laboratory equipment was delivered. In October 2014, LANL scientists traveled to Uganda to assist UVRI staff in setting up the genome center and to deliver the first round of on-site training to UVRI staff. LANL staff returned to UVRI in February 2015 to deliver additional training in laboratory protocols and bioinformatics analysis. During this visit, the genome center started sequencing and analyzing several clinical samples of unknown viral diseases.

#### Gabon: International Center for Medical Research in Franceville

Los Alamos National Laboratory is working with a research institute in Gabon, the International Center for Medical Research in Franceville (CIRMF) to enhance their genomics capabilities. CIRMF was founded in 1979 to conduct research in fertility, immunology, pathology, and microbiology. Today, CIRMF has refocused almost exclusively on infectious diseases with an emphasis on microbiological monitoring and assistance to public health. LANL is assisting the CIRMF to set up a genomics center to achieve these objectives. In February 2015, LANL staff traveled to Franceville to provide technical assistance to the facility and staff, to assist in addressing technical challenges in operating a newly acquired MiSeq, and to set up a new bioinformatics system that we assisted CIRMF in acquiring. In this engagement, the early evaluation and planning phases were unnecessary, as the CIRMF was already in the process of developing a genomics capability. An evaluation of the current state and the level of expertise were performed and recommendations for additional equipment and training were given. These recommendations are now being implemented.

### Initial Cooperative Research Highlights

Through these international partnerships, extensive collaboration planning and executions are being carried out. After 2–3 years of collaboration, research efforts have started producing initial results at various centers. A few examples are listed below:

#### CRISPR Analysis of *Yersinia pestis* Strains from Georgia

Three *Y. pestis* strains isolated from two historical natural plague sites of Georgia were analyzed based on their CRISPR (Clustered Regularly Interspaced Short Palindromic Repeats) features. In the *Y. pestis* genome, three CRISPR elements YPa, YPb, and YPc are found at three distinct loci and presently 137 spacer sequences are known (9). These CRISPR elements are highly correlated with the region and location of isolation, potentially providing important genotyping and evolutionary information, which could help to trace the source of outbreaks. The MiSeq platform at the Georgian NCDC Lugar Center was used to conduct WGS of the isolates. The Georgian scientists have gained sufficient analytical skills through training, and successfully applied the two bioinformatic pipelines that have been deployed (CLCBio Genomics Workbench and EDGE Bioinformatics) to perform the analysis.

#### MERS-CoV Patient Sample Analysis

In 2013, an outbreak of MERS-CoV occurred in the Middle East, including Jordan (10, 11). Two individuals with severe respiratory symptoms were suspected of having been infected by the virus were admitted to the King Abdullah University Hospital; the throat swabs were sent to PHBC for analysis. One of the patients did not survive after hospital admission. Total RNA was isolated by PHBC staff, and samples were sequenced with the MiSeq platform and supplemented with additional Illumina HiSeq sequencing. The presence of MERS-CoV virus was confirmed in both samples using NGS. Even though the samples were degraded, the virus was present in sufficient abundance for us to obtain the entire viral genome from one of the samples, revealing that it was related to the original outbreak in Saudi Arabia. This type of high-resolution analysis of clinical samples can now be achieved at JUST by the trained local scientists and laboratory staff. It will enable faster detection and more detailed tracking of future outbreaks, and reach further research objectives.

#### RNA Virus Sequencing and Analysis at the UVRI Genome Center

After two rounds of training, the UVRI staff analyzed several blood samples from patients presenting with various viral hemorrhagic fever (VHF) symptoms. RNA was extracted and converted to sequencing libraries for sequencing on the UVRI MiSeq instrument. The URVI scientists applied the standardized protocols received during the laboratory setup and training session to perform an initial quality control check, quantification, RNA sample preparation, and concentration measurement. Sequencing library preparation, validation, and quantification were performed using standard protocols provided by LANL, and NGS was performed on the UVRI MiSeq. The Ugandan scientists successfully applied the two bioinformatic pipelines that have been deployed (CLCBio Genomics Workbench and EDGE Bioinformatics) to perform the sequencing data analysis. While no hemorrhagic fever viruses were present in the samples, the analyses revealed some potential bacterial pathogens that may have caused the symptoms.

Our collaboration partners continue to actively explore the application of NGS to their local needs. Research ideas include the characterization of samples from long-term archives. This would include the application of NGS to the investigation of past unexplained illnesses, characterization of pathogens from geographical areas across different time periods, and possibly the identification of previously unknown pathogens to the local area. Studying the zoonotic diseases endemic to the partner countries is also of high interest. And many partners are interested in developing a quality reference database of pathogen strains within their purview. Application to clinical, animal, and agricultural samples is a common theme.

## Conclusion

Our experience in collaborating with our international partners has been very fruitful and rewarding. We have successfully assisted in the development of genome centers in four countries: Republic of Georgia, Kingdom of Jordan, Uganda, and Gabon. We have also established long-term partnerships with genomic laboratories in many other countries. Enthusiasm for utilizing NGS at each center continues to build, and several projects have begun. These early projects aim to detect and characterize pathogens from clinical, animal, or environmental samples.

These collaborative experiences have not gone without certain challenges. Some of the challenges have been of a logistical nature, such as acquiring reagents in a timely fashion, which is critical to the success of the research and training. On more than one occasion after reagents were ordered, the items were delivered after the manufacturer listed expiration date due to logistical delays. Proper infrastructure support is another main challenge for NGS capability, including environmental heating, cooling and ventilation. Stable and clean electrical power is very important and not always present. Reliable and efficient Internet access is required for NGS operation; Internet access is improving rapidly in all partner locations, but remains a point of concern.

Partner staff expertise and proficiency building is key to the success of these collaborations. Most scientists and technicians are experienced biologists but lack bioinformatics expertise. Training a biologist in the techniques of genome science is relatively straightforward but achieving informatics proficiency requires a basic starting level which is lacking. The partner countries typically do not have ready access to bioinformatics skills, and this continues to be a challenge. Our recent deployment of the EDGE bioinformatics tool has been an attempt to overcome this challenge, which was designed for general users without specialized bioinformatics training.

Sustainment of the genome science capabilities with our partners is a key concern. We continue to improve the expertise of these partners through training and scientific conference opportunities. Sustainable use of the established NGS technologies will be strengthened by performing regular research projects. These research projects will be funded by international sponsors, and will provide funds for reagents and research time by partner staff. Over time, this approach will enable the partners to exercise and develop genomic capabilities and continue on a path to sustainability. A recently funded research project with NCDC in Georgia resulted from a competitive proposal process is a positive example.

Development of genome centers in many countries around the World will not only help the local public health authorities identify and monitor disease outbreaks but will also enable true global biosurveillance at a high temporal, geographic, and information resolution (12–14). Considering the potential for the rapid spread of the known pandemic pathogens and future emerging ones, a network of genome centers that can rapidly provide high-resolution genomic data will help improve the speed and accuracy of outbreak detection and monitoring, and reduce the global threat from these pathogens. The speed and efficacy of mitigation will be largely increased by the ability to test the samples locally, without having to ship the sample long distances. This approach offers a large safety benefit, as clinical samples containing live pathogens do not have to be moved long distances or across borders, and potentially cause additional outbreaks. Successful scientific partnerships and sustainable technical capacity are essential to addressing complex global health security challenges to realize the One Health concept.

## Conflict of Interest Statement

The authors declare that the research was conducted in the absence of any commercial or financial relationships that could be construed as a potential conflict of interest.
